# Gastric corticotropin-releasing factor influences mast cell infiltration in a rat model of functional dyspepsia

**DOI:** 10.1371/journal.pone.0203704

**Published:** 2018-09-07

**Authors:** Shin-ichiro Hagiwara, Esha Kaushal, Sreenivasan Paruthiyil, Pankaj J. Pasricha, Burcu Hasdemir, Aditi Bhargava

**Affiliations:** 1 The Osher Center for Integrative Medicine, University of California, San Francisco, San Francisco, CA, United States of America; 2 Division of Gastroenterology & Hepatology, Johns Hopkins School of Medicine, Baltimore, MD, United States of America; 3 Department of OBGYN, University of California, San Francisco, San Francisco, CA, United States of America; Duke University, UNITED STATES

## Abstract

Functional gastrointestinal disorders (FGIDs) are characterized by dysregulated gut-brain interactions. Emerging evidence shows that low-grade mucosal inflammation and immune activation contribute to FGIDs, including functional dyspepsia (FD). Stress plays an important role in the onset of FD symptoms. In human subjects with FD, presence of gastric mast cells has been reported, but factors that influence mast cell infiltration remain uncharacterized. Corticotropin-releasing factor (CRF) initiates the body’s stress response and is known to degranulate mast cells. In this study, we delineated the role of the CRF system in the pathogenesis of FD in a rat model. Gastric irritation in neonate rat pups with iodoacetamide (IA) was used to induce FD-like symptoms. RNA interference (RNAi) was used to silence gastric CRF expression. Mast cell infiltrate in the stomach increased by 54% in IA-treated rats compared to controls and CRF-RNAi tended to decrease gastric mast cell infiltrate. Sucrose intake decreased in IA-treated rats and mast cell numbers showed a negative association with sucrose intake. IA treatment and transient silencing of gastric CRF increased hypothalamic CRF levels. In IA-treated rats, gastric levels of CRF receptor 2 (CRF_2_) decreased by ~76%, whereas hypothalamic CRF receptor 1 (CRF_1_) levels increased. Plasma levels of TNF-α showed a positive correlation with plasma CRF levels. Levels of phosphorylated p38 and ERK1/2 in the stomach showed a positive correlation with gastric CRF levels. Thus, CRF may contribute to low grade inflammation via modulating mast cell infiltration, cytokine levels, MAPK signaling, and the gut-brain axis.

## Introduction

Functional dyspepsia (FD) is a clinical syndrome characterized by pain or burning in the epigastrium, early satiety, fullness during or after meal, or a combination of these symptoms that involve the upper gastrointestinal tract, such as the stomach and the duodenum [1, 2]. FD affects about 20% of world-wide population [3]. As per the latest iteration of the Rome process, the Rome IV criteria, FD is subdivided into two distinct categories: post-prandial distress syndrome and epigastric pain syndrome. Post-prandial distress syndrome is defined as meal-induced dyspeptic symptoms, bothersome post-prandial fullness, and early satiety for at least 3 days per week. Epigastric pain syndrome is defined by symptoms that occur in between meals for at least one day per week [1, 4, 5].

Although the etiology and pathophysiology of FD are not fully understood, Rome IV criteria proposed the possible contribution of low-grade inflammation, alterations in the gut microbiome composition, and altered brain processing of pathophysiological symptoms [1, 6, 7]. These inclusions were based on several studies that demonstrate presence of low-grade mucosal inflammation and immune cell activation in association with impaired epithelial barrier function and aberrant neuronal sensitivity in human subjects with functional gastrointestinal disorders (FGIDs) [8, 9]. The mucosal inflammatory infiltrate in the intestines of FGIDs subjects consisted mainly of mast cells, eosinophils, and intraepithelial lymphocytes. In a recent meta-analysis, mast cell counts in the stomach were found to be increased in individuals with FD compared to healthy subjects [10].

The gut-brain axis is thought to be dysregulated in subjects with FGID and patients often suffer from mood disorders, such as increased anxiety [11–13]. Stressors are known to exacerbate FGID symptoms [14]. The perception of stress activates the hypothalamic-pituitary-adrenal (HPA) axis. HPA axis activation is triggered by the release of corticotropin-releasing factor (CRF) from hypothalamus, which acts on the anterior pituitary to release ACTH, which in turn acts on the adrenal cortex to release glucocorticoids (cortisol in humans and corticosterone in rodents). Peptide hormones CRF, three urocortins (UCN1-3), and two G protein-coupled receptors, CRF receptors 1 (CRF_1_) and 2 (CRF_2_) coordinate stress and immune responses [15–18]. In the gastrointestinal tract, local and transient inhibition of CRF using RNA interference (RNAi) ameliorated inflammation in a *Clostridium* Toxin A-mediated model of ileitis [19]. While UCN2-RNAi had no effect on inflammation in the ileitis model, both CRF and UCN2 modulated ileal motility [19]. Silencing of CRF in the colon also prevented stress-induced increases in fecal output, transepithelial conductance, and ion secretion [20].

A rat model for functional dyspepsia that uses early-life adverse events has been described [21]. In this model of functional dyspepsia, rats that experienced transient gastric irritation as neonates exhibited depression- and anxiety-like behaviors as adults. While central CRF- and vagal nerve-dependent mechanisms [21, 22] contributed to symptoms in FGID models, the role of gastric CRF in FD models has not been demonstrated. In this study, we elucidated the contribution of gastric CRF in functional dyspepsia symptoms and in modulation of the gut-brain axis in a rat model.

## Materials and methods

### Animals

All animal procedures were approved by the Institutional Animal Care and Use Committee (IACUC) at the University of California, San Francisco and were conducted in accordance with the National Institutes of Health Guide for the Care and Use of Laboratory Animals. All procedures were conducted in accordance to ARRIVE guidelines. Male Sprague-Dawley rats (Harlan/Envigo Laboratories, Indianapolis, IN) were used in all the experiments. The rats were housed in a room that was temperature (22–25°C) and light controlled (12-h:12-h light/dark cycle starting at 7:00 AM). They were handled daily to avoid handling act as a stressor. Pups were weaned at age of 3 weeks and were housed 2 pups per cage.

### Functional dyspepsia model

**Fig 1:**
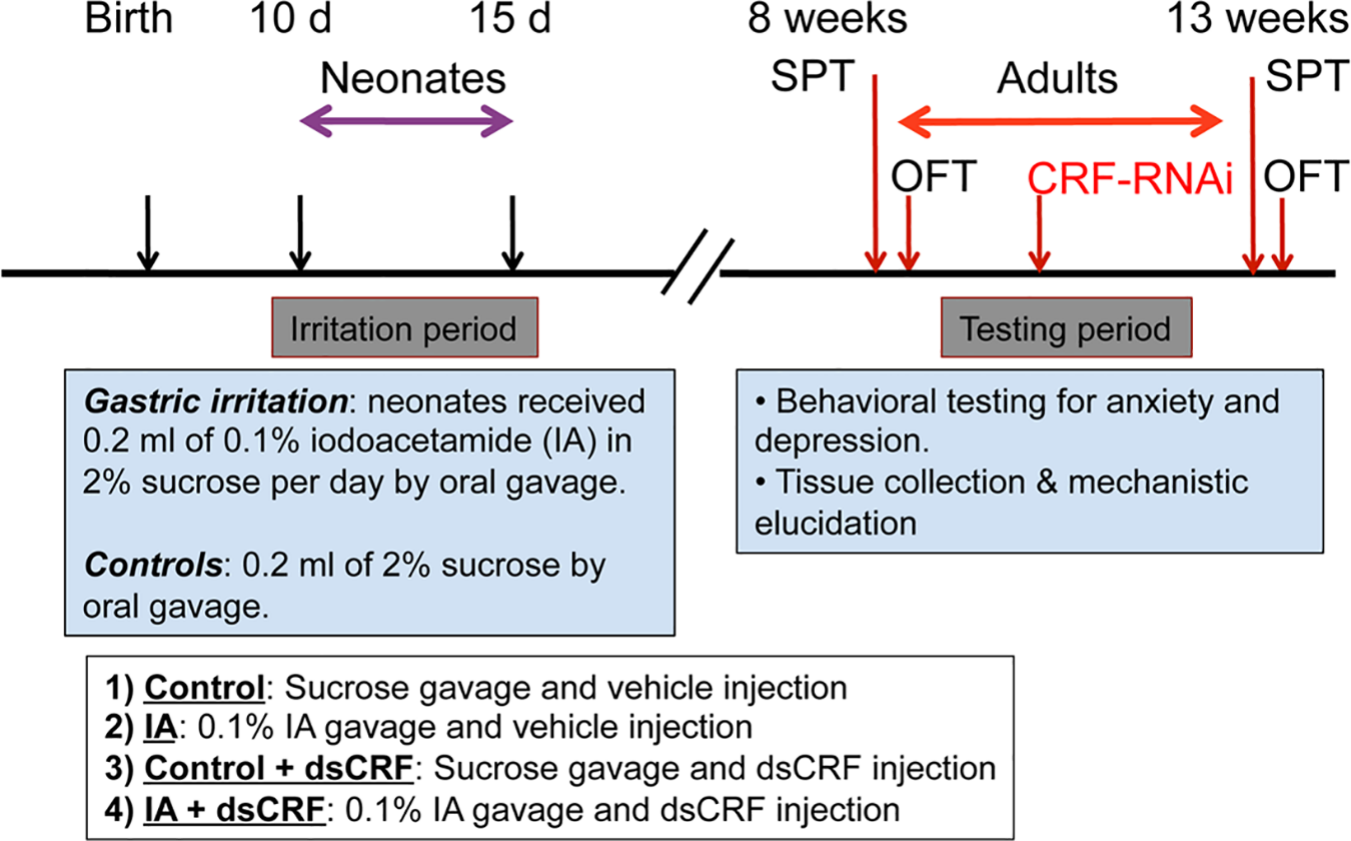
Schematic representation of FD model with time line for IA treatment, CRF-RNAi surgeries, and behavioral testing. To induce FD, ten-day old rat pups received IA or sucrose (control) by gavage. Eight weeks later, baseline behavioral tests (SPT and OFT) were conducted. Surgery for gastric RNAi injection (RNAi against CRF or vehicle control) was performed 1 week after behavioral testing and 5 days after surgery, behavioral tests (SPT and OFT) were repeated. Thus, the same rats served as their own baseline for behavioral testing. One week after surgery, rats were euthanized and various tissue samples harvested and blood collected. At 10-day of age, male rat pups were randomized to either control (2% sucrose gavage) or FD (0.1% IA gavage) groups (n = 16/group). At 8 weeks after gastric irritation, adult rats in control and FD groups were then randomized to receive the following surgical treatments: Group 1 (Control): Sucrose + vehicle injection (n = 8), Group 2 (IA): IA + vehicle injection (n = 8), Group 3 (Control dsCRF): Sucrose + dsCRF injection (n = 8), and Group 4 (IA dsCRF): IA + dsCRF injection (n = 8).

Functional dyspepsia model rat has been previously described [21]. Ten-day old rat pups received 0.2mL of 0.1% iodoacetamide (IA) in 2% sucrose daily for 6 days by oral gavage (performed by E.K and S.P). Control rat pups received 0.2mL of 2% sucrose alone. The pups were returned to their home cage with the dam after gavage and were weaned at 21 days. Rats were housed in pairs. At 8 weeks after IA or sucrose gavage, rats were randomized into four experimental groups to receive either double-stranded RNA against CRF (dsCRF) or vehicle (veh) in the stomach (Fig 1): Group 1 (Control): Sucrose + veh (n = 8), Group 2 (IA): IA + veh (n = 8), Group 3 (Control dsCRF): Sucrose + dsCRF (n = 8), and Group 4 (IA dsCRF): IA + dsCRF (n = 8). Rats were euthanized using isoflurane followed by decapitation to collect trunk blood for hormone assays. Hypothalami were dissected and flash-frozen in liquid nitrogen for protein/RNA analyses. Stomachs were removed, weighed, cleaned and re-weighed, cut longitudinally in half (to include both forestomach and corpus), and one half was fixed in 4% paraformaldehyde, and the other half frozen for protein/RNA analyses.

### Transient silencing of CRF in the stomach using RNAi

Surgery for RNAi injection was performed between 11–13 weeks after IA treatment (Fig 1). Briefly, rats were anesthetized with 2% isoflurane and 5% O_2_ using a nose cone and a vaporizer. A midline laparotomy was performed and the stomachs were exteriorized for injections. The exteriorized gut was kept moist with normal saline. Vehicle (groups 1 and 2) or 10 μg of dsCRF (groups 3 and 4) was injected intramuscularly at 4 or 5 sites into the stomach wall as described in our previous publications [19, 23, 24]. After injection, the abdominal fascia was closed in two layers. Rats were given one dose of buprenorphine as analgesics immediately after surgery and returned to home cage. Surgeries were performed by S-I.H and A.B.

### Behavioral testing

Behavioral tests were conducted in sequential order; sucrose preference test (SPT) was performed first, followed by open field test (OFT), with one- to two-day interval in between the tests. SPT and OFT were performed twice; once at 8–10 weeks after IA treatment, but before surgery and once after injection of vehicle or dsCRF (at least 3–5 days post-surgery) as delineated in Fig 1. Behavioral tests were performed by E.K.

### Sucrose preference test

Sucrose preference test was performed as described previously [21]. Briefly, rats were subjected to a 48-hour training session and a 1-hour test session conducted 24 hours after the training session. In the 48-hour training session, singly-housed rats were given a choice of two bottles, one bottle containing sucrose solution (1%, W/V) and the second bottle containing tap water. The bottles were placed to the left and right side of the feeding compartment, respectively and were switched every 12 hours to prevent possible effects of side preference in drinking behavior. After the training session, only tap water was provided for 6 hours. Food and water were then withheld from rats for 18 hours. Subsequently, in the test session, rats again had a choice of two bottles; one with sucrose solution (1%, W/V) and the other with tap water, respectively, for one hour. Sucrose intake was calculated as follows: sucrose intake (g) divided by body weight at the day of SPT [25].

### Open field test

The open field apparatus consisted of an arena (122 cm × 122 cm × 46 cm) made of rectangular black plastic [26–28]. Individual rats were gently placed into the center of the box and allowed to explore the arena for 30 minutes. Movements were recorded by a video camera mounted above the arena, and analyzed using TopScan Realtime option version 3.00 software (CleverSys Inc., Reston, VA). The videos were analyzed by the Gladstone’s Neurobehavioral Core (CA) by personnel who were blinded to the groups.

### Antibodies

Primary antibodies: The primary and secondary antibodies, dilutions used, and sources were as follows: Antibodies from Cell Signaling Technology, Danvers, MA: phospho-ERK (31912; mouse, 1:1,000) [29], phospho-p38 (#9211S; rabbit, 1:1,000) [15]; Santa Cruz Biotechnology, Santa Cruz, CA: CRF_1_ (sc-1238; goat, 1:200) [30], CRF_2_ (sc-20550; goat; 1:1,000) [15], ERK (sc154; rabbit, 1:1,000) [29], p38 (sc7972; mouse; 1:1,000) [15]; Thermo Fisher Scientific, Waltham, MA: phospho-JNK1/2 [Thr183, Tyr185] (44-682G; rabbit; 1:1,000) [31]; MilliporeSigma, Billerica, MA: JNK2 (05–986; mouse; 1:1,000) [32], β-actin (A2228; mouse; 1:5,000) [15]; Courtesy of Prof. W. Vale: CRF (rabbit; 1:5,000) were used. Secondary antibodies: For immunofluorescence staining goat anti-rabbit conjugated to Rhodamine Red-X (Jackson ImmunoResearch) at 1:500 dilution was used. For Western blot analyses donkey anti-goat/rabbit conjugated to Alexa Fluor 680 (Thermo Fisher Scientific) and donkey anti-mouse conjugated to IRDye 800 (Rockland Immunochemicals, Pottstown, PA) at 1:20,000 dilution were used.

### Western blot analyses

Stomach and hypothalamus tissue samples were homogenized in RIPA buffer (50 mM Tris-HCl, pH7.4, 150 mM NaCl_2_, 5 mM MgCl_2_, 1 mM EGTA, 10 mM NaF, 10 mM Na_4_P_2_O_7_, 0.5% Nonidet P-40; supplemented with protease inhibitor cocktail (Roche, Mannheim, Germany) and phosphatase inhibitor cocktails (Sigma-Aldrich). Lysates (40 μg) was boiled with Laemmli Sample Buffer (BIO-RAD, Hercules, CA), resolved with 10% SDS-PAGE, transferred to polyvinylidene difluoride membranes (PVDF, Immobilon-FL; Millipore, Billerica, MA) and blocked for 1 h at room temperature in Odyssey Blocking Buffer (Li-COR Biosciences, Lincoln, NE). Membranes were incubated with primary antibodies overnight at 4°C. Membranes were washed for 30 min (1 × PBS, 0.1% Tween20) and incubated with secondary antibodies for 1 h at room temperature. Blots were analyzed with the Odyssey Infrared Imaging System.

### Mast cell count

Longitudinal stomach sections (5μM thick) were stained with Toluidine blue. Mast cells were counted at ×20 magnification in 10 fields per forestomach and corpus area with a BZ-X700 microscope (KEYENCE, Osaka, Japan) and average mast cell number was calculated after pooling data from all sections/rat. Mast cells were counted by a single researcher (S-I. H) in a blinded manner. Images of the corpus and forestomach were acquired using a Nikon Eclipse bright field microscope (x20 and x40 objectives), and images were captured using AxioVision Imaging software.

### ELISA for mast cell Tryptase, CRF and Cytokines

Total protein concentration was measured using BCA assay (BIO-RAD). Tryptase levels in gastric lysates (90μg/well) were measured using a Rat Tryptase ELISA Kit (ABclonal, Woburn, MA). CRF levels in gastric (50μg/well) and hypothalamic lysates (45μg/well), and plasma (25μL/well) were measured using a Rat CRF ELISA Kit (Phoenix Pharmaceuticals Inc., Belmont, CA). Cytokines (TNF-α, IL-1β, IL-6, and IL-10) levels were measured in gastric lysates (30μg/well) and plasma (25μL/well) using a proinflammatory panel 2 V-PLEX Rat ELISA Kit (Meso Scale Discovery, Gaithersburg, MD) on the MSD platform. All kits were used according to the manufacturers’ instructions.

### Immunofluorescence and microscopy

Longitudinal stomach sections (5μM thick) were deparaffinized in xylene and rehydrated in ethanol series. Sections were incubated in blocking buffer (1x PBS, 0.3% Triton X-100, 10% normal goat serum) for 1 h at room temperature. Sections were incubated with primary antibody (anti-CRF) overnight at 4°C, washed, and incubated with fluorescent secondary antibodies (conjugated to Rhodamine Red-X) for 1 h at room temperature. Images were acquired using a Nikon Eclipse epi-fluorescence microscope (20x and 40x objectives) and images were captured using AxioVision Imaging software.

### Statistical analysis

Two-way ANOVA was used to analyze interactions between treatment groups. When main effects were significant, Student’s *t* test was used to compare two data points using Prism v7.0 software (GraphPad Software Inc., La Jolla, CA). A p value of p<0.05 was considered statistically significant and tendencies were discussed at p>0.051 to 0.40. Data are shown as mean ± standard error of the mean (SEM).

## Results

### Mast cell numbers increased in the stomach of IA-treated rats

**Fig 2:**
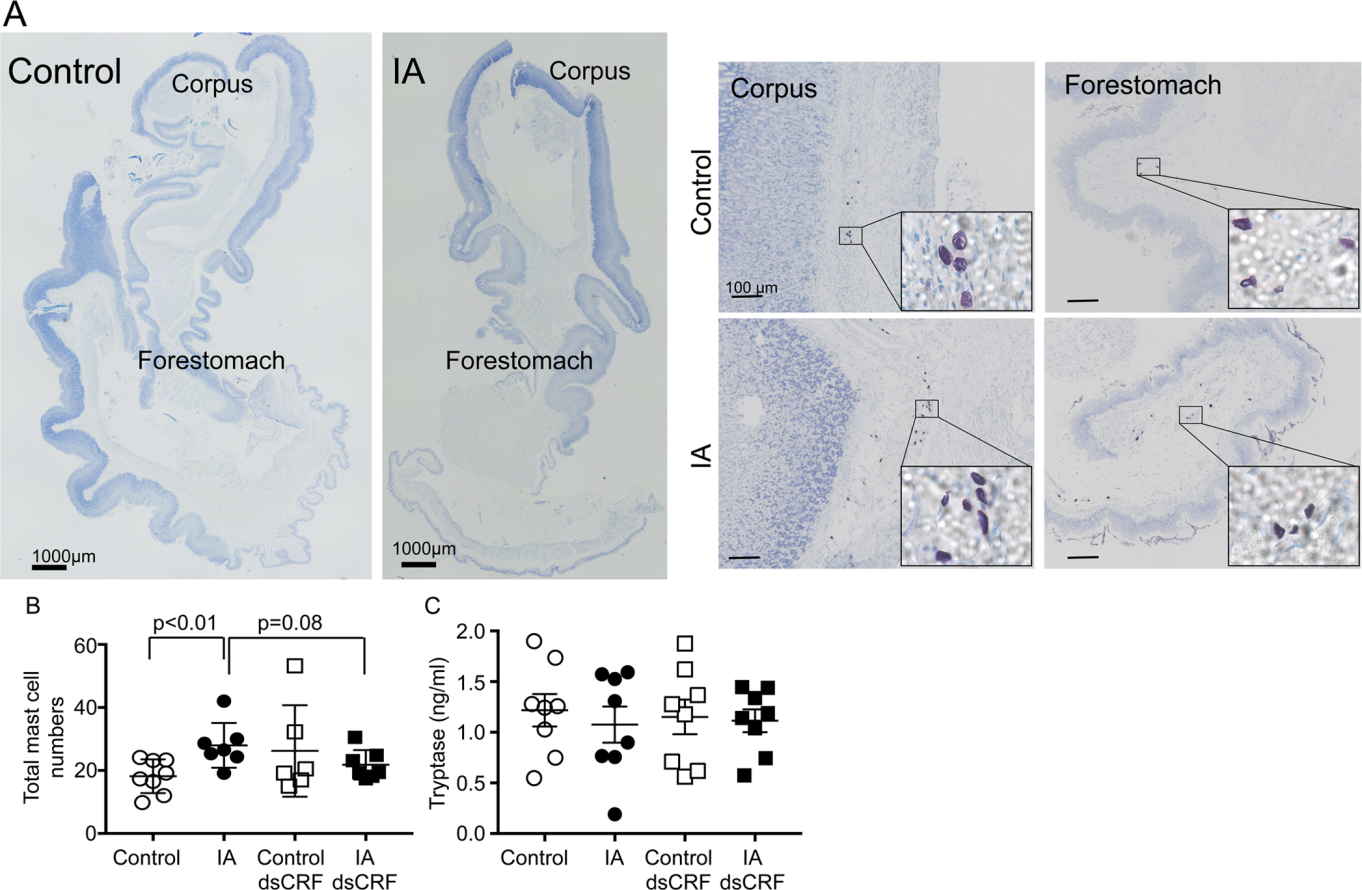
Modulation of mast cell infiltration in FD model. (**A)** Representative stitched images of toluidine blue-stained stomach sections of control and IA rats (scale bar = 1000μm). Magnified images of the corpus and the forestomach areas are shown (scale bar = 100μm); inset: zoomed-in mast cells. (**B)** Two-way ANOVA showed significant main interaction between IA treatment and CRF-RNAi injection (p<0.05). Mast cell numbers were higher in IA-treated rats than control rats (p<0.01; Student’s t-test; IA: 28.0 ± 2.7 vs. Control: 18.2 ± 1.9; n = 7-8/group). In IA-treated rats, dsCRF injection tended to decrease the numbers of mast cells compared with vehicle-injected controls (p = 0.08; Student’s t-test; IA: 28.0 ± 2.7 vs. IA dsCRF: 21.8 ± 1.8; n = 7-8/group). (**C**) Mast cell tryptase levels in gastric lysates did not differ between control and IA-treated groups.

Low-grade inflammation and presence of mast cells have been reported in human subjects with FD [10, 33], but have not been determined in animal models. Toluidine blue-stained stomach sections revealed that mast cell infiltration was present in the submucosal layers of corpus and forestomach regions (Fig 2A). Mast cell numbers increased by 54% in IA-treated rats compared to controls (Fig 2B, IA vs. Control, p<0.01). CRF is known to degranulate mast cells and thereby exacerbates inflammation [34]. While mast cell infiltration increased in the gastric mucosa of IA-treated rats, no significant change in mast cell degranulation was noted between IA-treated and control rats (Fig 2A, inset).

### CRF-RNAi in the stomach modulated mast cell numbers in IA-treated rats

We ascertained if transient inhibition of gastric CRF expression affected mast cell numbers or granulation. CRF-RNAi did not affect mast cell degranulation. Two-way ANOVA revealed significant main interaction between CRF-RNAi and IA treatment on mast cell numbers (F (1,24) = 4.945; p<0.05). CRF-RNAi in IA-treated rats tended to decrease the numbers of mast cells in the stomach compared with IA-treated rats (Fig 2B, IA vs. IA dsCRF, p = 0.08). Next we determined mast cell tryptase levels in gastric lysates. Neither IA nor CRF-RNAi treatments affected gastric tryptase levels (Fig 2C).

### Mast cell numbers showed an association with behavior measures

**Fig 3:**
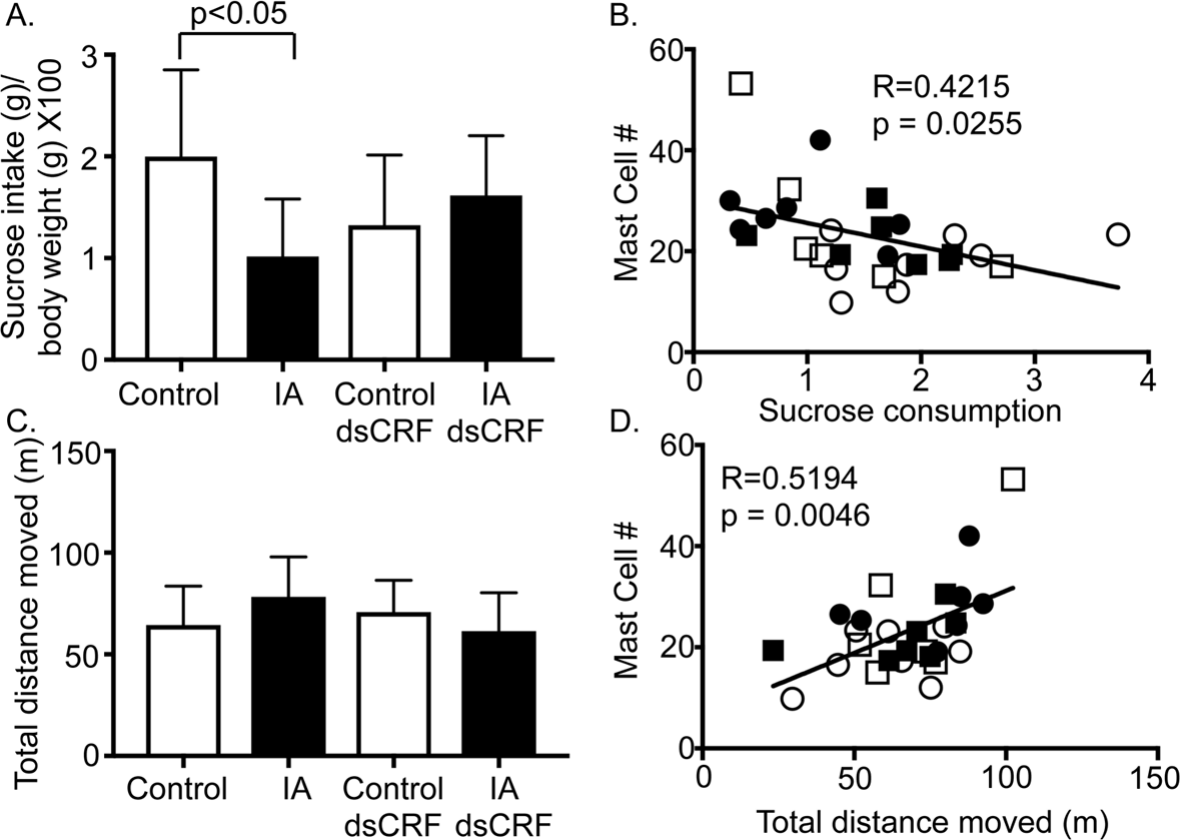
Behavioral modulation in FD by gastric CRF and association with mast cells. (**A)** Two-way ANOVA revealed significant main interaction between IA treatment and CRF-RNAi injection on sucrose intake (p<0.05). Sucrose intake of IA-treated rats (IA) significantly decreased compared with control rats (p<0.05; Student’s t-test; IA: 1.02 ± 0.2 vs. Control: 2.00 ± 0.30; n = 8/group). Transient silencing of gastric CRF tended to restore sucrose consumption or preference in IA-treated rats. (**B)** Linear regression analyses showed a negative correlation between mast cell numbers and sucrose consumption. (**C**) Neither IA treatment (IA) nor transient silencing of gastric CRF modulated anxiety-like behavior as reflected by unchanged total distance moved or thigmotaxis in open field test. (**D**) Linear regression analyses showed a positive correlation between mast cell numbers and total distance moved. Empty circles (Control), filled circles (IA), empty squares (Control dsCRF), and filled squares (IA dsCRF).

Decreased sucrose intake is reflective of anhedonia and IA-treatment has been shown to result in depression-like behavior in adult rats [21], but the role of CRF in mediating sucrose intake has not been shown. Two-way ANOVA revealed significant main interaction between IA treatment and CRF-RNAi on sucrose intake (F (1,28) = 6.961; p<0.05). Sucrose intake decreased in IA-treated rats compared to controls (Fig 3A, IA vs. Control, p<0.05). Since mast cells are known to modulate gut-brain function, we next determined whether mast cells showed any relationship with sucrose intake. Sucrose intake showed an inverse correlation with mast cell numbers in the stomach (Fig 3B, R = 0.4207; p = 0.0255).

Anxiety-like behavior is reported in animal models of functional bowel disorders [21, 35]. In this study, no significant main effect of IA treatment or CRF-RNAi was observed on the total distance traveled, thigmotaxis, or time spent in the center during 30-minute test period between groups (Fig 3C). However, mast cell numbers showed a positive association with total distance moved (Fig 3D, R = 0.5194; p = 0.0046).

### Transient-silencing of CRF increased gastric transit

**Fig 4:**
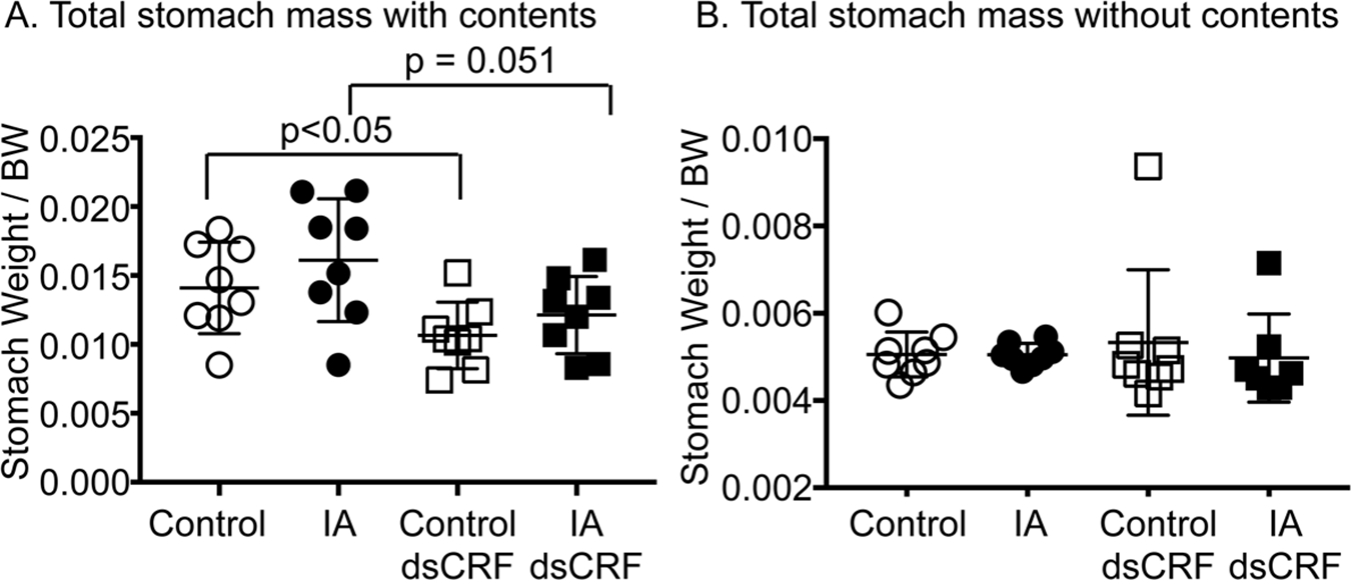
Transient silencing of gastric CRF altered gastric emptying. (**A)** Two-way ANOVA indicated significant effect of CRF-RNAi injection on stomach contents (p = 0.004). The total weight of the stomach with its content was lower in rats after CRF-RNAi (p<0.05; Student’s t-test; Control dsCRF: 0.0107 ± 0.0009 vs. Control: 0.0141 ± 0.0012; n = 8/group, p = 0.051; Student’s t-test; IA dsCRF; 0.0121±0.0009 vs. IA 0.01611±0.0015; n = 8/group). (**B)** The weight of empty stomachs was similar between the 4 groups.

Intraperitoneal or intravenous administration of CRF has been show to delay baseline gastric transit via activation of CRF_2_R in otherwise naïve animals, suggesting that CRF delayed gastric transit [36–38]. Two-way ANOVA showed main effect of CRF-RNAi on stomach contents (F(1,28) = 9.873; p = 0.004), but no effect of IA treatment. Transient silencing of CRF in control rats decreased gastric contents by 24.1% compared to vehicle-injected controls (Fig 4A, Control dsCRF vs. Control; p<0.05), suggesting that gastric transit increased. Similarly, CRF-RNAi decreased gastric contents in IA-treated rats by 24.7% compared to IA-treated rats (Fig 4A, IA dsCRF vs. IA, p = 0.051). Importantly, stomach mass without contents did not differ between groups (Fig 4B), suggesting that IA treatment did not alter baseline stomach size. Neither IA nor CRF-RNAi treatments affected body weights, colon length, and spleen weights.

### Transient-silencing of CRF altered hypothalamic CRF levels

**Fig 5:**
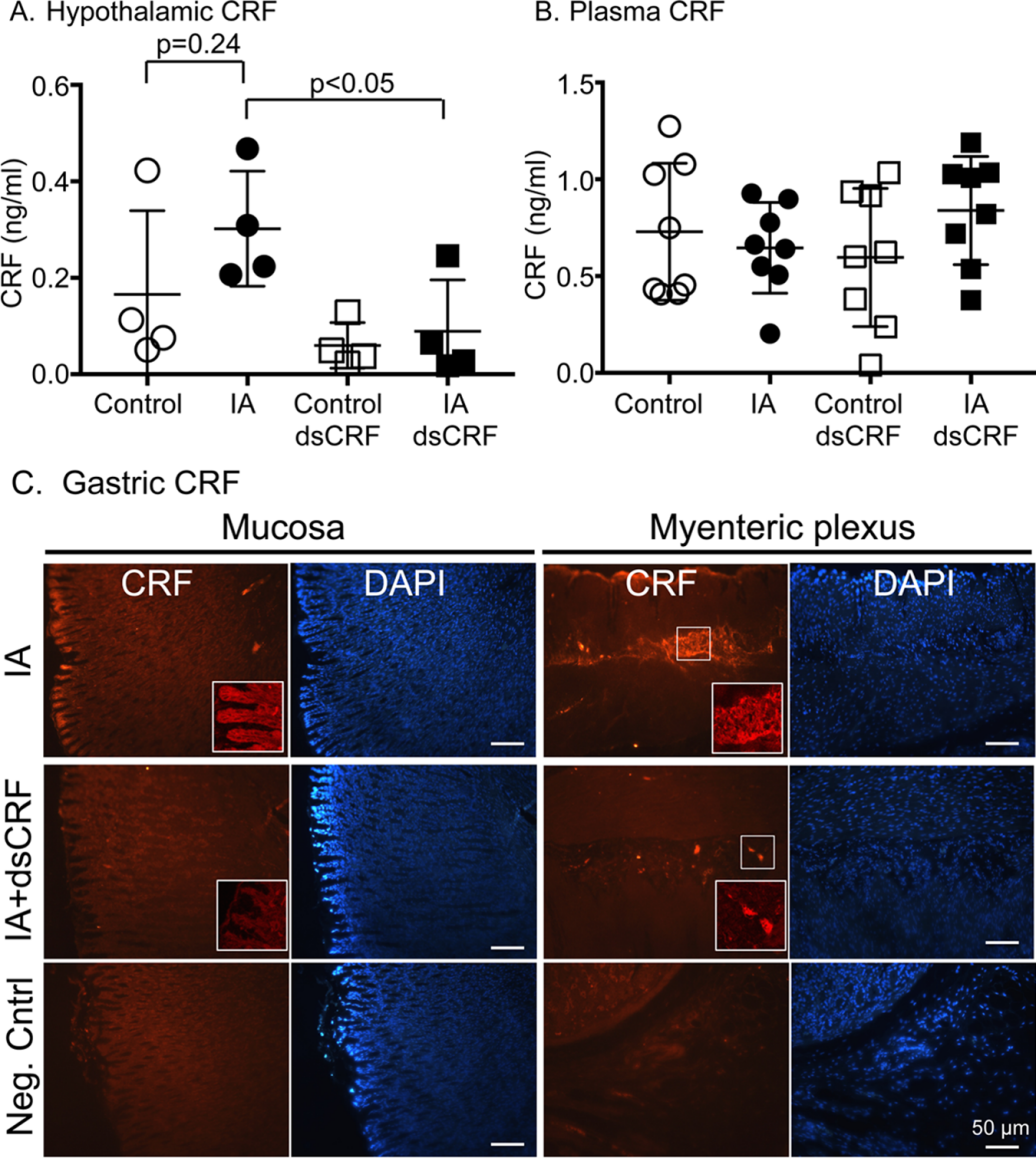
Gastric CRF modulated hypothalamic CRF levels. (**A**) CRF levels in the hypothalamus decreased after transient silencing of CRF in the stomach using RNAi in IA-treated rats (p<0.05; Student’s t-tests; IA dsCRF: 0.09 ± 0.05 vs. IA: 0.30 ± 0.06; n = 8/group) compared to control rats, reflecting a bidirectional feedback of the gut-brain axis. (**B**) Plasma CRF levels were not changed by either IA treatment or after transient silencing of CRF in the stomach. (**C**) Representative images of CRF-stained stomach sections of IA rats without (top row) and with (middle row) dsCRF treatment (scale bar = 50μm). Bottom row is negative control staining where primary antibody was excluded. Inset: Higher power (40x) images of the brush border of the mucosal layer and the myenteric neurons.

CRF released from the brain is known to mediate both central and peripheral effects, but it is not known if gastric CRF can feedback to mediate central effects. We previously showed that CRF immunoreactivity in the hypothalamic paraventricular nucleus (PVN) was increased in IA-treated rats [21]. Although IA treatment increased CRF levels in the hypothalamic lysates by 82% compared to controls, it did not attain statistical significance (Fig 5A, IA vs. Control, p = 0.24). IA treatment also resulted in non-significant increases in CRF levels in the stomach compared to controls (IA vs. Control, p = 0.37). Transient silencing of CRF in the stomach decreased CRF levels in the hypothalamus in IA-treated rats compared to vehicle-injected IA-treated rats (Fig 5A, IA dsCRF vs. IA, p<0.05). Neither IA nor CRF-RNAi treatments affected plasma CRF levels (Fig 5B). Robust CRF immunostaining was detected in the mucosal layers and myenteric neurons in stomach sections from IA-treated rats (Fig 5C, top row). In the presence of dsCRF, CRF signal was undetectable in the mucosal layer and the signal was very weak or patchy in the myenteric neurons (Fig 5C, middle row). No staining was detected when primary antibody was excluded (Fig 5C, bottom row).

### IA treatment and CRF-RNAi modulated CRF_2_ receptor levels

**Fig 6:**
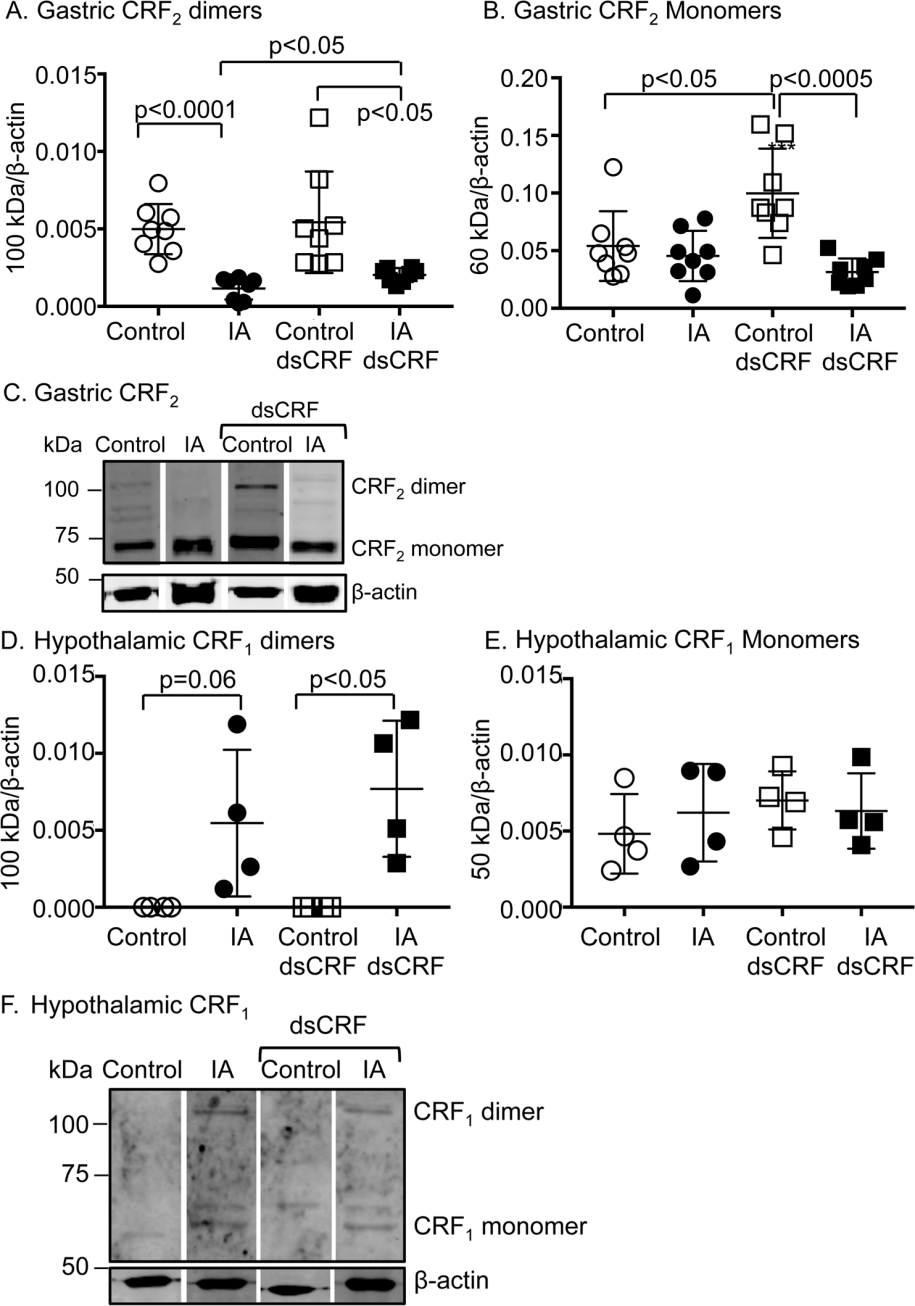
Modulation of CRF receptor levels in FD. Western blot analyses showing quantification of CRF receptor dimeric and monomeric bands in stomach and hypothalamic protein lysates. β-actin, a housekeeping gene was used as a loading control and its levels were used for normalization. (**A)** Two-way ANOVA showed significant effect of IA treatment on CRF_2_ expression levels in the stomach (p<0.0001). CRF_2_ dimer levels (~100 kDa band) decreased by ~76.8% in IA-treated rats compared to controls (p<0.0001; Student’s t-tests; n = 8/group). CRF-RNAi increased CRF_2_ dimer expression in FD rats (p<0.05; Student’s t-tests; n = 8/group), but not in control rats. (**B)** Two-way ANOVA revealed significant main effect of IA treatment and interaction between IA treatment and CRF-RNAi injection (p<0.001 and p<0.005, respectively). CRF_2_ monomer (~60 kDa band) expression increased in control rats after CRF-RNAi (p<0.05; Student’s t-test; n = 8/group), suggesting that ligand levels regulate receptor expression levels. (**C)** Representative Western blots showing gastric CRF_2_ mono- and dimers. (**D)** Two-way ANOVA revealed significant main effect of IA treatment on CRF_1_R levels in the hypothalamus (p<0.005). CRF_1_ dimer (~100 kDa band) expression increased in IA-treated rats compared to control- rats (p = 0.06; Student’s t-tests; n = 4/group). CRF-RNAi further increased CRF_1_ dimer levels in IA rats (p<0.05; Student’s t-tests; n = 4/group). (**E**) CRF_1_ monomer levels (~50 kDa band) in the hypothalamus were not affected by either IA or CRF-RNAi treatments. (**F)** Representative Western blots showing hypothalamic CRF_1_ monomers and dimers.

CRF_2_ is predominantly expressed in peripheral tissues including in the gastrointestinal tract and mediates anti-inflammatory effects [15, 39, 40]. CRF_2_ can exist as both monomers and/or (hetero)dimers [41, 42]. Two-way ANOVA showed significant main effect of IA treatment on expression of CRF_2_ dimers (F(1,28) = 29.8; p<0.0001). Expression levels of CRF_2_ dimers decreased by 76.8% in stomachs of IA-treated rats compared to controls (Fig 6A, IA vs. Control; p<0.0001). CRF-RNAi did not affect baseline expression levels of CRF_2_ dimers (Fig 6A, Control dsCRF vs. Control). CRF_2_ dimer levels remained lower by 53.6% in IA-treated rats with CRF-RNAi compared with control rats with CRF-RNAi (Fig 6A, IA dsCRF vs. Control dsCRF; p<0.05), but transient silencing of CRF in the stomachs of IA-treated rats increased CRF_2_ dimer levels by 66.6% compared to vehicle-injected IA-treated rats (Fig 6A, IA dsCRF vs. IA; p<0.05).

Two-way ANOVA also showed significant main effect of IA treatment (F(1,28) = 15.7; p<0.001) and interaction between IA treatment and CRF-RNAi (F(1,28) = 9.48; p<0.005) on expression levels of CRF_2_ monomer band (Fig 6B and 6C). CRF-RNAi increased CRF_2_ monomer levels by 84.8% compared to vehicle-injected controls (Fig 6B, Control vs. Control dsCRF; p<0.05), suggesting that transient silencing of gastric CRF affected expression levels of its receptor, CRF_2_. After CRF-RNAi in IA-treated rats, CRF_2_ monomer levels were reduced by 68.6% (Fig 6B, Control dsCRF vs. IA dsCRF; p<0.0005).

Central effects of CRF are mediated via activation of CRF_1_. Two-way ANOVA showed main effect of IA treatment on expression of CRF_1_ dimers (F(1,12) = 16, 41; p<0.005). CRF_1_ dimer levels increased in hypothalamus of IA-treated rats and transient silencing of CRF further increased CRF_1_ dimer levels in IA-treated rats (Fig 6D, Control dsCRF vs. IA dsCRF; p<0.05). Neither IA nor CRF-RNAi treatments affected CRF_1_ monomer levels (Fig 6E and 6F). Of note, CRF_1_ levels were not detected in stomach lysates and conversely, CRF_2_ levels were undetected in hypothalamic lysates.

### CRF levels are positively associated with TNF-α levels

**Fig 7:**
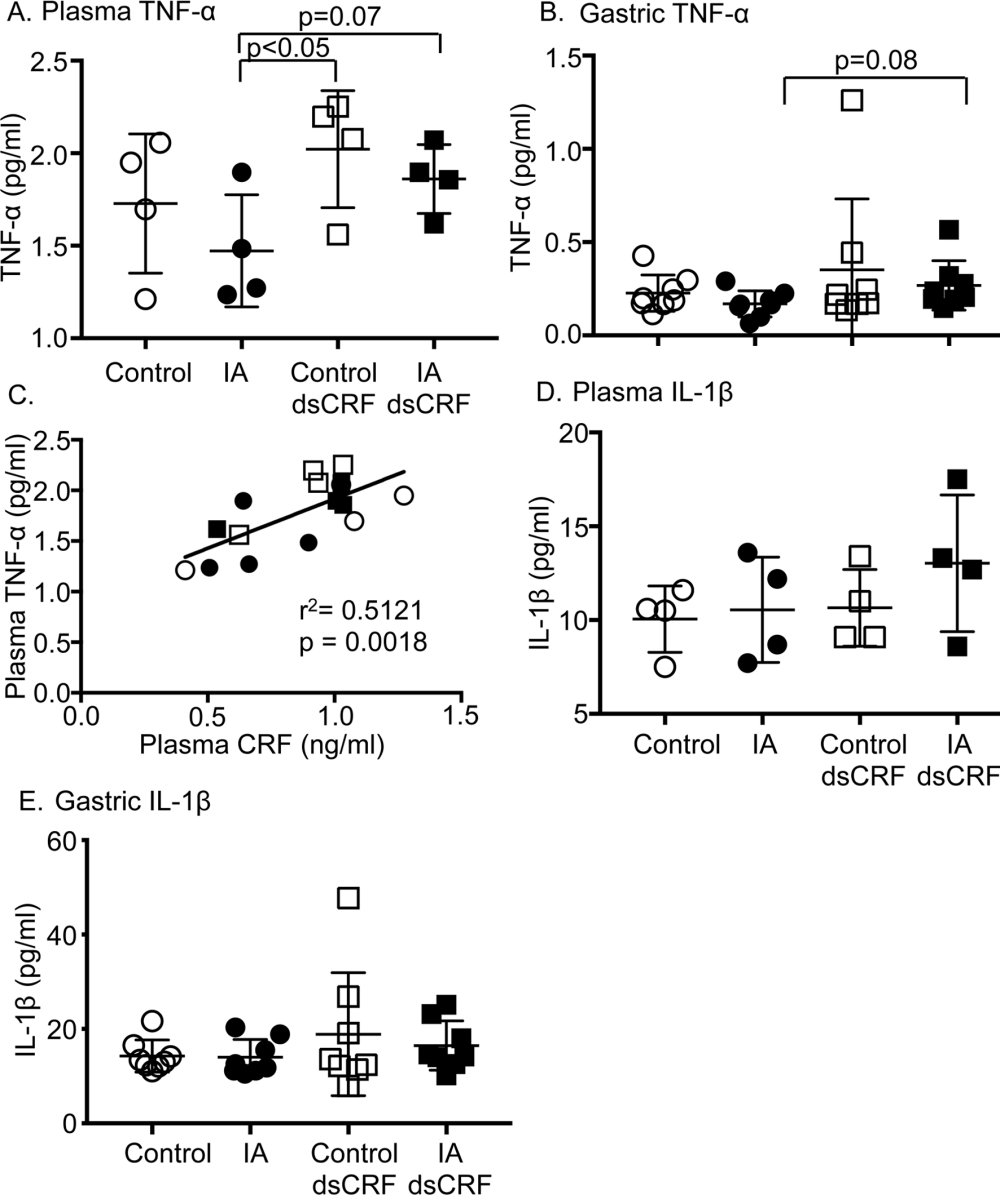
Modulation of cytokine levels in FD. (**A)** Two-way ANOVA indicates significant main effect of CRF-RNAi on plasma TNF-α levels (p<0.05). Plasma TNF-α levels increased after CRF-RNAi treatment (p = 0.07; Student’s t-test; IA: 1.47　±　0.15 vs. IA dsCRF: 1.86 ± 0.09; n = 4/group and p< 0.05; Student’s t-test; IA: 1.47 ± 0.15 vs. Control dsCRF: 2.02 ± 0.16; n = 4/group). (**B)** Gastric TNF-α levels tended to increase after CRF-RNAi in IA rats (p = 0.08; Student’s t-test; IA dsCRF: 0.27 ± 0.04 vs. IA: 0.17 ± 0.02; n = 8/group). (**C)** Linear regression analyses showed that plasma TNF-α levels were directly proportional to plasma CRF levels. Empty circles (Control), filled circles (IA), empty squares (Control dsCRF), and filled squares (IA dsCRF). (**D)** Plasma IL-1β levels (n = 4/group) and (**E)** gastric IL-1β showed no significant differences between the 4 groups (n = 8/group).

The involvement of cytokines in FD remains equivocal. In this rat model of FD, IA-treatment did not affect plasma or gastric TNF-α levels compared to controls (Fig 7A and 7B). Transient silencing of CRF increased baseline plasma TNF-α levels in IA-treated rats as well as in controls compared to vehicle-injected IA-treated rats (Fig 7A, IA vs. IA dsCRF; p = 0.07 and IA vs. Control dsCRF; p<0.05). Gastric TNF-α levels tended to increase in IA-treated rats with CRF-RNAi compared to IA-treated rats that had vehicle injection (Fig 7B, IA dsCRF vs. IA, p = 0.08). Plasma CRF levels showed a strong positive correlation with plasma TNF-α levels (Fig 7C, R = 0.7155; p = 0.0018). Plasma IL-1β levels tended to be higher in IA-treated rats after RNAi, but gastric IL-1β levels did not differ between groups (Fig 7D and 7E). Plasma and gastric IL-6 and IL-10 levels were below detection limits in all 4 groups.

### IA treatment and gastric CRF modulated MAPK signaling

**Fig 8:**
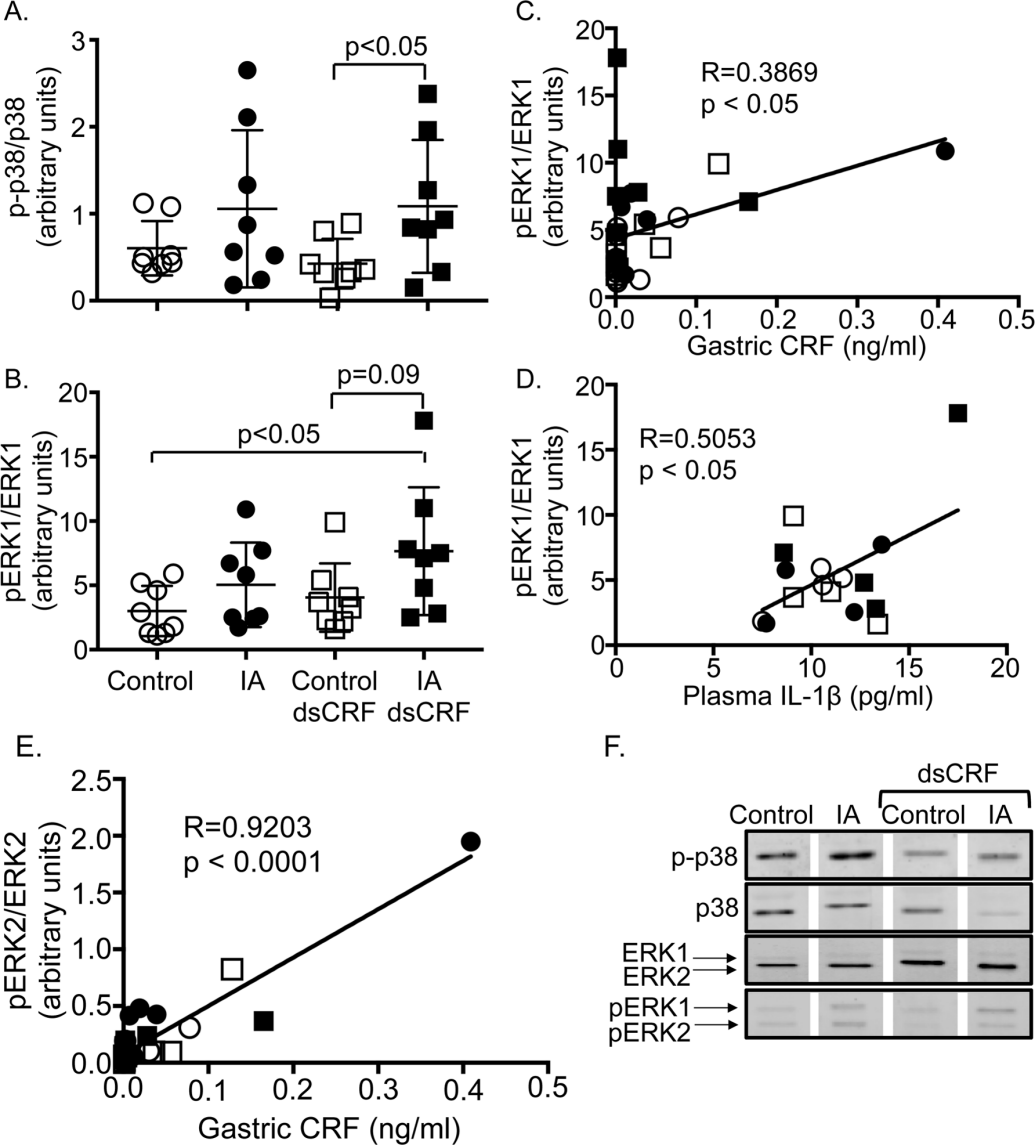
Modulation of phosphorylation of MAPK in FD. Western blot analyses showing quantification of phosphorylated levels of p38 and ERK1/2. Total levels of p-38 and ERK1/2 were used for normalization. (**A)** Two-way ANOVA showed significant main effect of IA treatment on p-p38 levels (p = 0.018). Levels of p-p38 increased in IA-treated rats compared to controls after CRF-RNAi (p<0.05; Student’s t-tests; n = 8/ group). (**B)** Two-way ANOVA revealed a significant main effect of IA treatment on pERK1 levels (p = 0.026). Levels of pERK1 increased in IA-treated rats after CRF-RNAi compared to controls (p = 0.09; Student’s t-tests; n = 8/group). (**C)** Linear regression analyses showed that the levels of pERK1 in the stomach were directly proportional to the CRF levels in the stomach (R = 0.3869, p<0.05). (**D)** Linear regression analyses showed that the levels of pERK1 correlated directly to the plasma levels of IL-1β (R = 0.5053, p<0.05). (**E)** Linear regression analyses showed that levels of pERK2 in the stomach were directly proportional to gastric CRF levels (R = 0.9192, p<0.0001). Empty circles (Control), filled circles (IA), empty squares (Control dsCRF), and filled squares (IA dsCRF). (**F**) Representative Western blots showing gastric p-p38, p38, ERK1/2, and p-ERK1/2 levels.

MAPK signaling is known to regulate cytokine production in models of inflammation, but the involvement of MAPKs in FD model is unknown. We ascertained the effect of IA treatment and CRF-RNAi on phosphorylation levels on p-38, ERK1/2, and JNK. Two-way ANOVA showed main effect of IA treatment on expression of phospho-p38 in gastric lysates (F(1,28) = 6.272; p = 0.018). CRF-RNAi treatment significantly increased gastric p-p38 levels by 155% in IA-treated rats compared to controls (Fig 8A and 8F, IA dsCRF vs. Control dsCRF, p<0.05). Further analysis showed that phospho-p38 levels did not show any significant association with mast cell numbers, CRF, TNF-α, or IL-1β levels (gastric or plasma).

Two-way ANOVA showed main effect of IA treatment on expression of phospho-ERK1 in the stomach (F(1,28) = 5.492; p = 0.026). Phospho-ERK1 (pERK1) levels increased in the gastric lysates by 89.2% in IA-treated rats compared to controls after CRF-RNAi (Fig 8B and 8F, IA dsCRF vs. Control dsCRF, p = 0.09). pERK1 levels were also significantly increased by 154% in IA-treated rats with transient silencing of CRF compared with controls (Fig 8B IA dsCRF vs. control, p<0.05). Gastric CRF (Fig 8C, R = 0.3869; p<0.05) and plasma IL-1β levels (Fig 8D, R = 0.5053; p<0.05) displayed a positive relationship with p-ERK1 levels. Mast cell numbers or TNF-α levels did not exhibit any association with gastric pERK1 levels. While p-ERK2 levels did not differ between groups after IA treatment or CRF-RNAi, gastric CRF levels showed a strong positive correlation with pERK2 levels (Fig 8E and 8F, R = 0.9203; p<0.0001). Mast cell numbers or IL-1β levels did not exhibit any association with p-ERK2 levels. Phospho-JNK levels were not affected by either IA treatment or CRF-RNAi nor showed any association with mast cell numbers, CRF, TNF-α, or IL-1β levels in the stomach or plasma.

## Discussion

**Fig 9:**
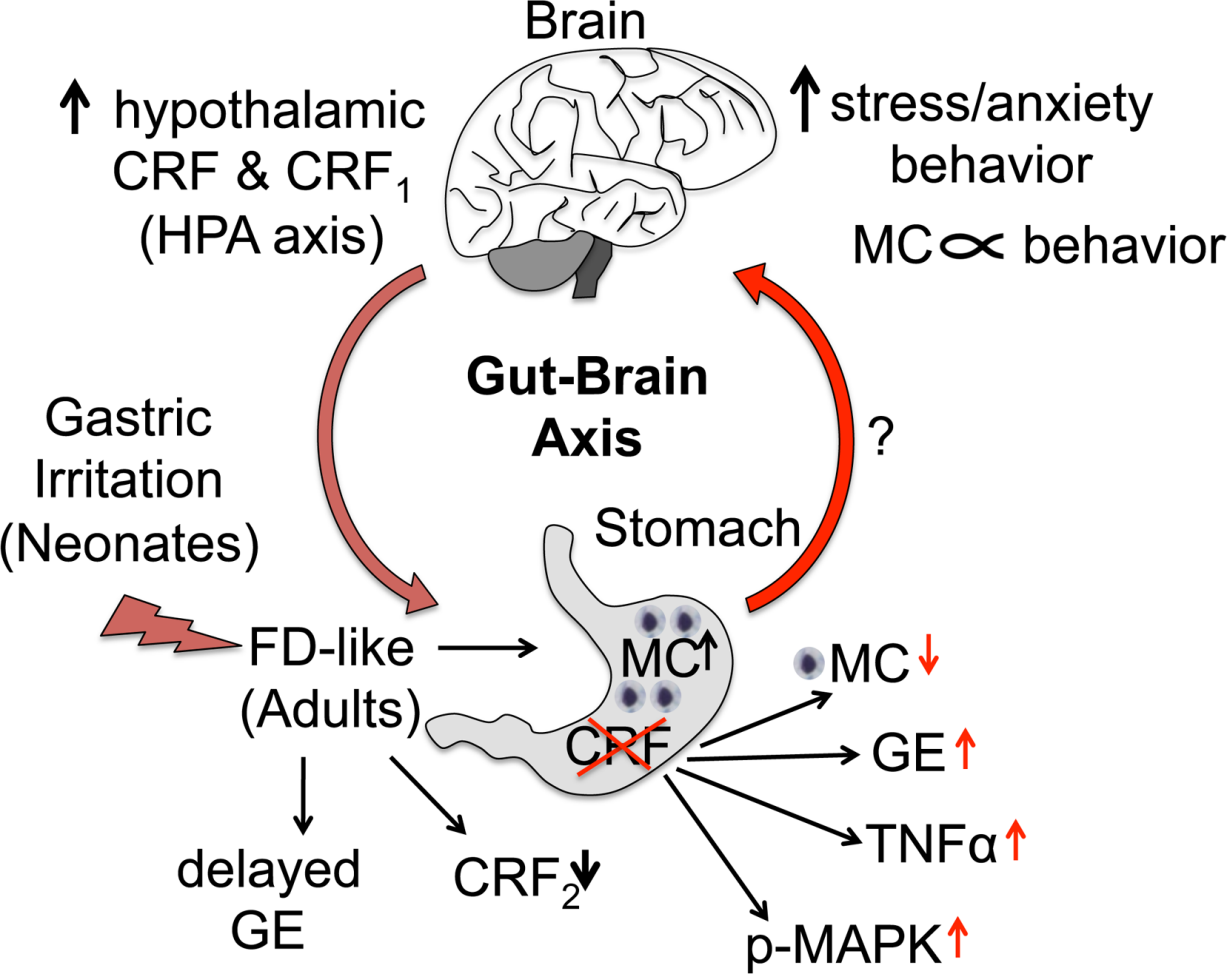
Gut-brain axis modulation by gastric CRF: Schematic showing summary of findings. Gastric irritation in the neonatal period increased mast cell infiltration, delayed gastric emptying, decreased CRF_2_ expression in the stomach, and increased hypothalamic CRF and CRF_1_ expression in adult rats. IA treatment modulated depression-like behavior, which also correlated negatively with mast cell numbers. Anxiety-like behavior, on the other hand, correlated positively with gastric mast cell numbers. Taken together, these data suggest a dysregulated gut-brain feedback that may involve gastric mast cells. Silencing of gastric CRF led to decreased numbers of gastric mast cells, and restored gastric transit. CRF levels were directly proportional to TNF-α and pERK1/2 expression levels in the stomach. Gastric silencing of CRF prevented IA-induced increases in hypothalamic CRF levels, suggesting that gastric CRF feeds back to the hypothalamus, which has been postulated for patients with FGID, but has not been demonstrated so far (as depicted by a question mark). Thus, a bidirectional gut-brain information flow may be involved in the pathogenesis of FD. CRF: corticotropin-releasing factor; CRF_1_: CRF receptor 1; CRF_2_: CRF receptor 2; FD: functional dyspepsia; GE: gastric emptying; HPA axis: hypothalamic-pituitary-adrenal axis; MC: mast cell.

Low-grade inflammation and a dysregulated gut-brain axis are being recognized as important contributors to FGID symptoms. Early-life adverse events are a risk factor for development of FGID in adulthood [43]. In this study, using a previously described rat model of FD where 10-day old neonates were exposed to gastric irritation over a period of several days, we found that mass cell infiltrate increased in stomachs of IA-treated adult rats and associated with behavioral changes in adult rats. Both IA treatment and transient silencing of gastric CRF expression altered hypothalamic CRF and CRF_1_ expression. Behavioral changes are modulated by activation of the HPA axis via hypothalamic CRF and CRF_1_, thus gut-brain feedback appears to be modulated by gastric CRF as summarized in Fig 9. Interestingly, CRF levels displayed a direct relationship with TNF-α as well as with phosphorylation status of several MAPK levels, suggesting that several immune and cell signaling pathways are modulated by CRF.

Mast cells are potentially important effectors in causation of FGID symptoms, including in FD subjects. They are found in proximity to nerves in locations throughout the body, including the gut mucosa [8, 44]. Mast cell numbers were increased in the antrum and corpus regions of the stomach in *Helicobacter pylori*-negative FD subjects [45, 46]. In addition, a recent meta-analysis study showed that mast cell numbers were greater in the stomachs of FD patients compared to healthy subjects [10]. Our finding of increased mast cell numbers in the whole stomach in IA-treated rats is consistent with findings in human FD patients and confirms that early-life adverse events can lead to presence of low-grade inflammation that may persist in adulthood. In mast cell deficient mice and blockade of brain, but not peripheral mast cells, was shown increase anxiety- and depression-like behavior in mice [47, 48]. Thus, in our study, it is possible that modulation of gastric CRF might have altered brain mast cells numbers along with gastric mast cell numbers, thereby altering behavior.

CRF is known to induce degranulation of mast cells and thereby exacerbates inflammation [34]. However, the source of gastric CRF is not known. Since RNAi works at the mRNA level, our CRF-RNAi in the stomach suggested that stomach cells also locally transcribed CRF. Transient silencing of CRF in the stomach tended to decrease mast cell numbers in IA-treated rats (Fig 9), but did not alter mast cell degranulation, raising the possibility that locally expressed CRF influences mast cell infiltration, but may not be sufficient by itself to activate mast cells.

Psychological distress, including anxiety and depression, is associated with FD [12, 49]. IA-treated rats showed depression-like behavior. Others also reported depression- and anxiety-like behaviors in animal models of FD [20, 21, 43]. In this study, both anxiety-and depression-like behavior measures were associated with mast cell numbers, albeit in an opposite manner. Depression-like behavior was negatively associated with mast cell numbers, whereas anxiety-like behavior was positively associated. While others have shown that in rat models of FD, the amount of time spent in the center or open arm was less in behavioral tests [21], we did not find any difference between IA-treated and control rats in the amount of time spent in the center or thigmotaxis. This discrepancy can be explained by the sex of the researcher performing behavioral tests. It was reported that male researchers increased anxiety-like behavior in rodents, whereas female researchers did not [50]. In our study, all behavioral training, testing, and animal care were performed by one woman researcher (E.K) and female husbandry staff. This may account for the differences reported in other FGID models using behavioral measures as well. Nonetheless, in our study, total distance moved showed a direct relationship with mast cell numbers present in the stomachs of rats. Thus, reducing chronic mast cell or other immune cell infiltration may have beneficial effect on FGID symptoms. In agreement with our findings, treatment with mast cell stabilizer, disodium cromoglycate, suppressed the effect of acute psychological stress on small bowel permeability in human subjects [51].

Psychological factors are important symptoms in FGIDs [52]. While a dysregulated gut-brain axis is closely linked to psychological morbidity in FGIDs [53], bidirectional feedback of the gut-brain axis has not been demonstrated unequivocally. Here, we show that hypothalamic CRF and CRF_1_ expression levels increased in IA-treated rats compared to controls, suggesting that early-life adversity, such as gastric irritation, modulated hypothalamic CRF signaling via the gut-brain axis. Our novel finding that silencing of gastric CRF prevented IA-induced increases in hypothalamic CRF, strongly suggests a bidirectional gut-brain communication may be involved in the pathogenesis of FD.

Accumulating evidence demonstrates that peripheral CRF is involved in the regulation of gastrointestinal motility. Our observations revealed that transient silencing of gastric CRF expression decreased gastric contents in general or increased gastric transit. In agreement with these observations, others and we have previously reported that CRF modulates gastric emptying and bowel motility [19, 20, 22]. Astressin, a nonselective CRF antagonist delayed gastric transit and blocked CRF and stress-induced gastric contractions in guinea pigs and rats [36, 54]. In agreement with these published data, we found a significant interaction between IA-treatment and CRF-RNAi on expression levels of CRF_2_ in the stomach. While both monomers and dimers of CRF_2_ were detected in the stomach, IA-treatment appeared to specifically reduce dimer expression. Receptor dimerization can modify the functional characteristics of the CRF receptors, including downstream signaling as we have described previously [41]. Whether a balance between CRF receptor monomer/hetero(di)mer alters signaling in gastric cells under pathophysiology remains to be determined. Post-translational modifications of receptors could be another contributing mechanism for altered function of the CRF system in functional bowel disorders [42, 55]. In the periphery, CRF_2_ is the predominant receptor and pharmacological inhibition of its function is known to block CRF- and stress-induced delayed gastric emptying [56]. Thus, increase in local CRF levels due to stress or inflammation may delay gastric motility and/or transit.

MAPKs signaling are activated by CRF receptors via changes in intracellular Ca^2+^ and cAMP levels. p38 plays a key role in regulating TNF-α in primary human macrophages [57] and in regulating cytoprotective Hsp27 [15, 58]. MAPKs regulate heat shock proteins [15], NF-κB [59], and other target proteins to increase expression of a plethora of proinflammatory cytokines, that include TNF-α, IL-1β, and IL-6. We investigated a subset of MAPKs whose phosphorylation levels may be regulated by altered CRF/CRF receptor levels and signaling. Others and we have previously shown that phosphorylation of MAPK including p38 and ERK1/2 is altered in experimental models of gastrointestinal inflammation [29, 60, 61]. In this study, IL-6 and IL-10 levels were undetectable in serum or gastric lysates, thus these cytokines do not appear to contribute to FD symptoms in this model. However, their role in human FGID cannot be ruled out. Transient silencing of gastric CRF increased phspho-p38 levels in IA-treated rats. CRF levels were directly proportional to the phosphorylation levels of p-ERK1/2. CRF levels were positively associated with TNF-α levels, suggesting that increased CRF levels may contribute to low-grade inflammation by increasing TNF-α levels. Furthermore, p-ERK1 levels were positively associated with IL-1β, suggesting that p-ERK affected IL-1β expression.

Functional dyspepsia, a relapsing and remitting disorder, is the most common cause of symptoms in the epigastric region and the upper gastrointestinal tract [2]. Stress is a major contributing factor for FGID symptoms, including FD, likely through dysregulation of the brain-gut axis. In this study, we showed that in a rat model of FD, gastric CRF is key modulator of the gut-brain axis and contributes to FD symptoms by mechanisms that include mast cells.
